# Cytoprotective Effects of Citicoline and Homotaurine against Glutamate and High Glucose Neurotoxicity in Primary Cultured Retinal Cells

**DOI:** 10.1155/2017/2825703

**Published:** 2017-10-15

**Authors:** Sergio Davinelli, Flavia Chiosi, Roberto Di Marco, Ciro Costagliola, Giovanni Scapagnini

**Affiliations:** Department of Medicine and Health Sciences “V. Tiberio”, University of Molise, Campobasso, Italy

## Abstract

Citicoline and homotaurine are renowned compounds that exhibit potent neuroprotective activities through distinct molecular mechanisms. The present study was undertaken to demonstrate whether cotreatment with citicoline and homotaurine affects cell survival in primary retinal cultures under experimental conditions simulating retinal neurodegeneration. Primary cultures were obtained from the retina of fetal rats and exposed to citicoline plus homotaurine (100 *μ*M). Subsequently, neurotoxicity was induced using excitotoxic levels of glutamate and high glucose concentrations. The effects on retinal cultures were assessed by cell viability and immunodetection of apoptotic oligonucleosomes. The results showed that a combination of citicoline and homotaurine synergistically decreases proapoptotic effects associated with glutamate- and high glucose-treated retinal cultures. This study provides an insight into the potential application of citicoline and homotaurine as a valuable tool to exert neuroprotective effects against retinal damage.

## 1. Introduction

Visual impairment is a worldwide health problem affecting about 285 million people [1]. Recently, it was estimated that with aging populations in high-income regions of Central/Eastern Europe, diabetic retinopathy and glaucoma will become the most important causes of vision loss [2]. The key cell type implicated in the development of glaucoma and diabetic retinopathy is the retinal ganglion cell (RGC), and apoptosis of RGC is the final event leading to visual loss [3, 4]. The cause of apoptosis is excitotoxicity due to excessive synaptic glutamate activity. Glutamate is one of the major excitatory neurotransmitters in the brain and exists in high concentrations in the retina. It is thought that exposure to moderately elevated levels of glutamate can trigger cellular processes in neurons that eventually lead to apoptosis [5, 6]. In addition, increasing evidence shows that several neuronal cell types in the retina are highly susceptible to hyperglycemia-mediated apoptosis [7]. Cell culture models have substantially contributed to the characterization of the pathophysiology of retinal neurodegeneration, providing a simplified tool to investigate in an isolated context the detrimental effects of high glucose (HG) concentrations and an excessive amount of glutamate [8, 9]. In recent years, research efforts have been made to identify neuroprotective drugs able to prevent visual field loss and preserve visual function. However, the failure of recent clinical trials raised several doubts regarding the strategies to achieve neuroprotection in retinal degeneration [10]. Based on the results of the latest investigations, it is reasonable to assert that a single constituent that affects one target has limited efficacy in preventing the progression of multifactorial diseases. A large body of research revealed that the use of a combination of compounds with synergistic multitarget effects may offer a more powerful approach for disease prevention, including retinal neurodegeneration [11–15]. This study investigated whether cotreatment of citicoline and homotaurine exhibits synergistic neuroprotective effects in experimental conditions associated with neuroretinal degeneration such as glutamate-induced excitotoxicity and HG-induced neurotoxicity. Citicoline (cytidine-5′-diphosphocholine) is an intermediate in the synthesis of phosphatidylcholine, a component of cell membranes. It has been shown that citicoline produces neuroprotective effects in a variety of central nervous system (CNS) injury models, particularly cerebral ischemia [16]. At the experimental level, it has been reported that citicoline is a neuroprotective molecule acting through mechanisms relevant to glaucoma and diabetic retinopathy. The effects proposed to explain the neuroprotective actions of citicoline have been thoroughly reviewed and include antiapoptotic effects, neurotrophic properties, protection after partial optic nerve crush, reduction of excitotoxicity, effects on nonglutamatergic neurotransmitter systems, and effects on remyelination [17]. In recent human studies, citicoline appears to be a promising agent to improve cognitive impairment [18]. Homotaurine (3-aminopropanesulfonate), an analogue of 4-aminobutyrate (*γ*-aminobutyric acid, GABA), is a small natural aminosulfonate compound identified in different species of marine red algae and then chemically synthesized and introduced into clinical use under the name of tramiprosate [19]. It has been shown that homotaurine may interfere with several cellular pathways, both *in vitro* and *in vivo* experimental models, and exert neuroprotective and neurotropic activities through different mechanisms including effects against the oxidative damage to DNA, antifibrillogenic activity, and antinociceptive and analgesic activities. More interestingly, beyond its neuroprotective and neurotropic effects related to the activation of GABA type A receptors, it has been observed that homotaurine prevents the neurotoxicity of A*β* peptide by reducing amyloid aggregation [20, 21]. Considering the distinct chemical properties of citicoline and homotaurine, the purpose of this study was to assess whether cotreatment of these compounds may exert synergistic neuroprotective effects on primary retinal cultures.

## 2. Materials and Methods

### 2.1. Retinal Cultures

The study has been approved by the appropriate ethics committee and has therefore been performed in accordance with the ethical standards laid down in the 1964 Declaration of Helsinki and its later amendments. Primary cultures were obtained from the retinas of fetal Wistar rats (18-19 days' gestation), following a procedure described elsewhere [22]. Briefly, retinal tissues were mechanically dissociated, and the cell suspensions were plated into 60 mm dish (0.8–1.0 × 10^6^ cells/mL) (Corning, Acton, MA). Retinal cultures were incubated in Eagle's minimal essential medium (MEM) containing 2 mM glutamine, penicillin-streptomycin (100 U/ml, 50 *μ*g/mL), and 25 mM N-(2-hydroxyethyl) piperadine-N′-(2-ethanesulfonic acid) (HEPES) under an atmosphere of 5% CO_2_ in the air. The medium was supplemented with 10% heat-inactivated fetal bovine serum during the 1st week and with 10% horse serum for the remaining 8–11 days. To eliminate nonneuronal cells, 10 *μ*M cytosine arabinoside (Sigma, St. Louis, MO) was added to the culture. Only those cultures maintained for 9–11 days *in vitro* and only isolated cells were used in this study. Previous studies using cultured rat retinal cells demonstrated that cell viability was reduced by exposure to glutamate (1 mM) for 10 min. Followed by postincubation in a glutamate-free medium for more than 1 hour [23, 24]. It was also showed that there was no significant difference between the values of reduction in cell viability between 1-hour and 24-hour incubations [25]. In the present study, glutamate neurotoxicity was assessed using a 25 min exposure to 100 *μ*M glutamate followed by a 24-hour incubation in the glutamate-free medium. In the second series of experiments, the cells were treated with HG concentrations to mimic the diabetic condition and produce a hyperglycemic insult. When cells reached 80% confluence, the culture medium was supplemented with glucose, reaching a final concentration of 30 mM. Retinal cells were exposed to HG for 96 hours. The concentration of glucose in control conditions was 5 mM. Media were changed every 24 hours in all groups.

### 2.2. Cell Viability

The assay used to assess cell viability in retinal cells was the (3,4,5-dimethylthiazol-2-yl)-2,5-diphenyltetrazolium bromide (MTT) reduction assay modified from that of Mosmann [26]. To evaluate the effect of citicoline and homotaurine on cell survival, the cells were subdivided into three groups and treated for 24 hours with 1 *μ*M, 10 *μ*M, and 100 *μ*M of citicoline (Kyowa Hakko Bio Co. Ltd., Tokyo, Japan) and with 1 *μ*M, 10 *μ*M, and 100 *μ*M of homotaurine (Truffini e Reggè Farmaceutici, Milan, Italy). To evaluate the neuroprotective effects of citicoline and homotaurine, cells were treated with citicoline 100 *μ*M, homotaurine 100 *μ*M, or citicoline + homotaurine 100 *μ*M, 24 hours before glutamate treatment and 30 min before HG treatment. MTT was added to wells at a final concentration of 0.5 mg/mL for 1 hour at 37°C. After this time, the medium was removed and reduced MTT (blue formazan product) was solubilized by adding 100 *μ*L dimethyl sulfoxide to each well. After agitation of plates for 15 min, the optical density of the solubilized formazan product in each well was measured using an automatic microplate reader (Molecular Devices, Crawley, UK) with a 570 nm test wavelength and a 690 nm reference wavelength.

### 2.3. Apoptotic Cell Death Detection

Apoptosis was determined by using a Cell Death Detection ELISA^PLUS^ kit (Roche Applied Science, Indianapolis, IN). This photometric enzyme immunoassay provides the quantitative determination of oligonucleosomes generated from the apoptotic cells. After the treatments, cells were washed, harvested, lysed, and centrifuged to remove nuclei, and supernatants were collected. An aliquot of the supernatant from each sample was incubated with immunoreagents in 96-well streptavidin-coated plates on a shaker. After three washes with incubation buffer, the substrate solution was added to each well, and absorbance was read at 405 nm in a microplate reader. The enrichment of oligonucleosomes released into the cytoplasm was calculated as absorbance of sample cells/absorbance of control cells.

### 2.4. Statistical Analysis

Data are expressed as the mean ± standard error of the mean (SEM) of three independent experiments. Statistical significance was determined using one-way analysis of variance (ANOVA), followed by Tukey's post hoc test. The *p* value < 0.05 was considered statistically significant.

## 3. Results

### 3.1. Viability of Primary Retinal Cultures Is Not Affected by Treatment with Citicoline or Homotaurine

**Figure 1:**
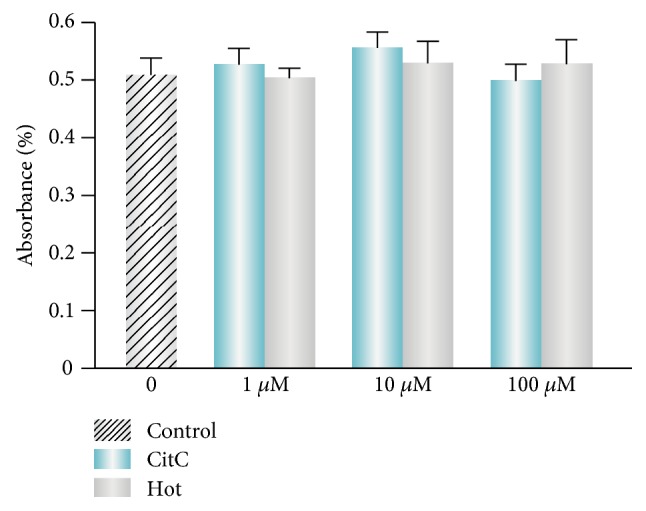
Effects of citicoline and homotaurine on cell viability. MTT assay shows that retinal cells were well preserved in citicoline- or homotaurine-treated cultures, with no evidence of toxicity after treatment at 1, 10, or 100 *μ*M. All data are represented as the mean ± SEM of three independent experiments. CitC: citicoline; Hot: homotaurine.

To determine the potential neuroprotective activity of citicoline and homotaurine, we firstly treated retinal cells with increasing concentrations of citicoline or homotaurine for 24 hours. We investigated whether 1 *μ*M, 10 *μ*M, and 100 *μ*M of citicoline or homotaurine may contribute to a reduced cell viability in retinal cells. As shown in Figure 1, retinal cells were well preserved in citicoline- or homotaurine-treated cultures, with no evidence of toxicity or significant loss of viability after treatments. Moreover, it has been previously shown that 100 *μ*M of citicoline is not harmful to retinal neuroglial cells *in vitro* and 100 *μ*M of homotaurine is an effective concentration to enhance neuroprotection in a model of experimental glaucoma [27, 28]. Therefore, this concentration of citicoline and homotaurine was used for all subsequent experiments.

### 3.2. Cotreatment of Citicoline and Homotaurine Exerts Synergistic Effects against Excitotoxic Cell Damage

**Figure 2:**
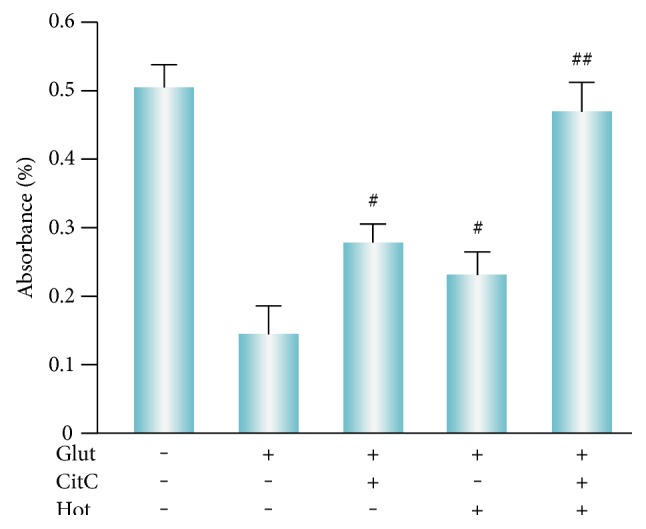
Cotreatment of citicoline and homotaurine protects retinal cells against glutamate-induced cytotoxicity. MTT assay was performed to detect cell viability after treatment with CitC and Hot against glutamate-induced cytotoxicity in retinal cells. The combined administration of citcoline and homotaurine demonstrated a significant synergistic cytoprotective effect. The results represent the mean ± SEM of three independent experiments. ANOVA followed by Tukey's post hoc test was carried out to determine the level of significance. ^#^*p* < 0.001 versus glutamate. ^##^*p* < 0.001 versus citicoline and homotaurine alone. CitC: citicoline; Hot: homotaurine; Glut: glutamate.

To evaluate whether cotreatment with citicoline and homotaurine was able to induce a synergistic neuroprotective effect against glutamate excitotoxicity, retinal cell cultures were exposed to citicoline 100 *μ*M, homotaurine 100 *μ*M, and citicoline + homotaurine 100 *μ*M, 24 hours before glutamate treatment. In the presence of 100 *μ*M citicoline, a significant increase in cell viability was observed (Figure 2). Although less effective than citicoline in terms of increased cell viability, significant neuroprotection was also observed following treatment with 100 *μ*M homotaurine (Figure 2). These data are consistent with previous studies, suggesting the neuroprotective activities of these compounds when used alone [28, 29]. However, the combination between citicoline and homotaurine significantly increased the viability of retinal cells after glutamate exposure (Figure 2). These results showed that combined administration of citicoline and homotaurine possesses a cytoprotective activity greater than the response achieved by the single compounds.

### 3.3. Cotreatment of Citicoline and Homotaurine Reduces Apoptosis Induced by Administration of Glutamate and HG

**Figure 3:**
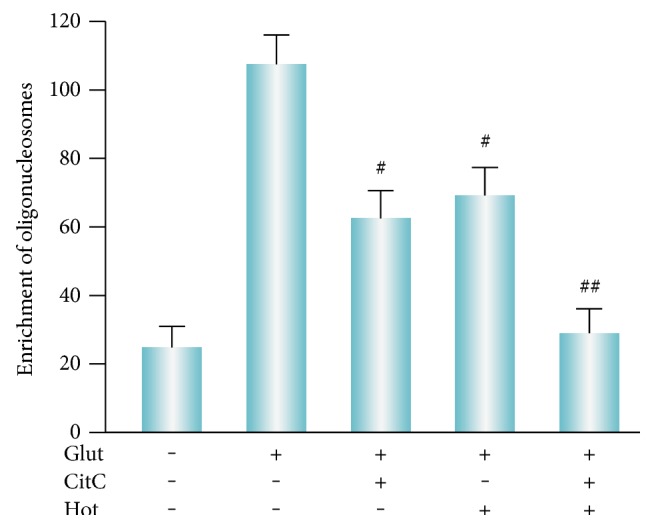
Cotreatment of citicoline and homotaurine significantly reduces the apoptotic rate in glutamate-treated cells. A cell death detection ELISA kit was used to determine cytoplasmic DNA oligonucleosome fragments associated with apoptotic cell death. The enrichment of oligonucleosomes released into the cytoplasm was calculated as absorbance of sample cells/absorbance of control cells. The administration of citicoline plus homotaurine demonstrated a synergistic effect in terms of apoptosis reduction. The results represent the mean ± SEM of three independent experiments. ANOVA followed by Tukey's post hoc test was carried out to determine the level of significance. ^#^*p* < 0.001 versus glutamate. ^##^*p* < 0.001 versus citicoline and homotaurine alone. CitC: citicoline; Hot: homotaurine; Glut: glutamate.

**Figure 4:**
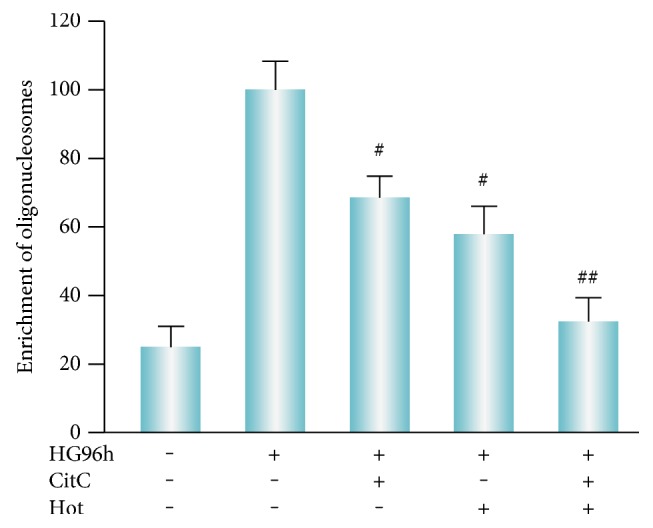
Combined administration of citicoline and homotaurine significantly reduces the apoptotic rate in high glucose-treated cells. Oligonucleosome fragments associated with apoptosis were quantified by cell death detection ELISA. As compared to the untreated cells, the administration of citicoline plus homotaurine demonstrated a statistically significant effect in terms of reduction of oligonucleosome levels. Data of three independent experiments are expressed as mean ± SEM of the absorbance from treated cells relative to absorbance of untreated cells. Comparison between data sets was performed using ANOVA followed by Tukey's post hoc test. ^#^*p* < 0.001 versus high glucose 96 hours. ^##^*p* < 0.001 versus citicoline and homotaurine alone. CitC: citicoline; Hot: homotaurine; HG: high glucose.

Next, we determined whether the synergistic effect of citicoline and homotaurine is associated with cytoprotection against glutamate-induced apoptosis. Apoptosis, measured by the number of oligonucleosomes released, was significantly decreased in cells incubated only with 100 *μ*M of citicoline 24 hours before glutamate treatment (Figure 3). Homotaurine 100 *μ*M also was able to decrease the neurotoxic effect glutamate in terms of reduction in apoptotic rate (Figure 3). However, as shown in Figure 3, reduction of retinal cell apoptosis induced by these compounds in combination was higher than the groups of either citicoline or homotaurine treated alone. These data suggest that citicoline or homotaurine in combination synergistically reduces apoptosis in glutamate-treated retinal cells. In addition, a neuroprotective effect was also observed against apoptosis induced by HG treatment. Primary retinal cell cultures, exposed to HG treatment, showed an increase in apoptosis, which was reduced in the presence of 100 *μ*M citicoline (Figure 4). Significant neuroprotective effects on apoptosis induced by HG treatment were also reported following treatment with homotaurine 100 *μ*M (Figure 4). Again in the presence of cotreatment with citicoline and homotaurine, apoptosis was significantly reduced in retinal cell cultures exposed to HG toxicity (Figure 4). Collectively, these results suggested that the enhanced reduction of apoptosis by combination treatment with citicoline and homotaurine may be a useful approach to exert a neuroprotective activity under conditions inducing retinal neurodegeneration.

## 4. Discussion

In this study, we tested synergistic neuroprotective effects of citicoline and homotaurine in combination on primary retinal cells exposed to glutamate toxicity and HG levels. The data demonstrated that cotreatment of citicoline and homotaurine has a direct neuroprotective effect in an experimental model of retinal neurodegeneration. Glutamate-induced excitotoxicity is implicated in the pathophysiology of several degenerative diseases of the retina, including glaucoma. Moreover, HG-induced neurotoxicity is a characteristic of diabetic retinopathy [30, 31]. Thus, the results of our study provide a rationale for the use of citicoline and homotaurine as potential therapeutic compounds in acute and chronic neurodegenerative diseases of the retina. To our knowledge, this is the first report demonstrating that the neurotoxic effect of glutamate and HG is greatly reduced by simultaneous application of citicoline and homotaurine. Therefore, the neuroprotective activity observed here provides also evidence that combinatorial treatment with these compounds may be a promising strategy to support retinal health. Indeed, an emerging therapeutic approach to counteract neuronal vulnerability associated with aging involves the mixture of distinct compounds, in order to improve the neuroprotective efficacy and pharmacokinetic-pharmacodynamic properties [32, 33]. Although the neuroprotective mechanisms of citicoline have been shown in various experimental models of retinal degeneration [34], the presence of homotaurine may increase the neuroprotective effects exerted by this compound. Moreover, it should be highlighted that recent studies have associated the neuroprotective activity of citicoline to its ability in activating sirtuin-1 (SIRT1), a member of the mammalian sirtuins important for neuronal plasticity, cognitive functions, as well as protection against aging-associated neuronal degeneration, and cognitive decline [17, 18]. The clinical efficacy of homotaurine has been extensively studied in several randomized, double-blind, placebo-controlled phase I, II, and III clinical trials, showing significant positive effects on secondary endpoints in patients with Alzheimer's disease [35, 36]. More interestingly, the association of homotaurine, carnosine, and forskolin (*Coleus forskohlii* root extract) has shown synergistic neuroprotective effects on RGC both *in vitro* and *in vivo* in a mouse model of hypertensive retinal ischemia [23, 37]. Although more than one mechanism might account these synergistic properties, this neuroprotection was associated with reduced calpain activity, upregulation of the phosphoinositide 3-kinase (PI3K)/Akt pathway, and inhibition of glycogen synthase kinase-3*β* (GSK-3*β*). Moreover, a recent pilot study demonstrated that oral administration of homotaurine, forskolin, carnosine, and folic acid improves intraocular pressure in patients with primary open-angle glaucoma [38]. Therefore, a multitarget approach by using a combination of molecules may be a more promising strategy to prevent retinal degeneration or slow down glaucomatous progression. In several experimental models of glaucoma, abundant evidence has been provided in which that RGC apoptosis is the earliest form of cell loss of the disease [39, 40]. Our results show that, following exposure to toxic levels of glutamate and glucose, cotreatment of citicoline and homotaurine reduced apoptosis of primary retinal cells (Figures 3 and 4). Although the neuroprotective mechanism of action of citicoline and homotaurine is not clear at this time, other authors have observed that particularly citicoline may reduce the retinal neuronal apoptosis induced by HG, increasing the expression of endogenous trophic factors such as brain-derived neurotrophic factor (BDNF) and ciliary neurotrophic factor (CNTF) that are transiently upregulated as part of the retinal defense responses. In addition, these effects were associated with the reduction of the expression of active forms of caspase-9 and caspase-3 [41, 42]. Alternatively, considering that toxic levels of glutamate and glucose induce an oxidative stress, by increasing reactive oxygen species (ROS), our findings may be also correlated with citicoline and homotaurine antioxidant activities [43, 44]. It should be also mentioned that taurine (2-aminoethanesulfonate), homotaurine analogue and one of the most abundant free amino acids in the brain, has been shown to attenuate retinal glial apoptosis in diabetic rats, suggesting an antiapoptotic action. It has also been suggested that taurine prevents glutamate excitotoxicity by increasing glutamate transporter expression, thereby decreasing glutamate levels. In diabetic patients, taurine depletion may be responsible for glaucomatous optic neuropathy, since RGCs are highly dependent on taurine for survival [45–48]. Altogether, the data presented here strongly suggest that citicoline and homotaurine in combination could be a potential new strategy for the prevention and treatment of neurodegenerative diseases, including glaucomatous retinopathy.

## 5. Conclusions

In conclusion, the present study demonstrated that cotreatment of citicoline and homotaurine exhibited synergistic neuroprotective effects on well-known experimental conditions of retinal neurodegeneration. Further studies are needed to clarify the mechanisms responsible for the observed neuroprotective properties, although our data suggest a reduction of apoptosis. Finally, these findings also suggest that cotreatment of citicoline and homotaurine may represent an interesting strategy to achieve neuroprotection in retinal neurodegeneration.
